# Hydrochar from Carbon Quantum Dots (CQDs) Synthesis for Photocatalytic and Decontamination Applications in Presence of TiO_2_

**DOI:** 10.3390/ijms26104958

**Published:** 2025-05-21

**Authors:** Daniel López, Karol Zapata, Lilian D. Ramírez-Valencia, Esther Bailón-García, Francisco Carrasco-Marín, Agustín F. Pérez-Cadenas, Camilo A. Franco, Farid B. Cortés

**Affiliations:** 1 Grupo de Investigación en Fenómenos de Superficie-Michael Polanyi, Departamento de Procesos y Energía, Facultad de Minas, Universidad Nacional de Colombia—Sede Medellín, Medellín 050034, Colombia; dalopezsu@unal.edu.co (D.L.); fbcortes@unal.edu.co (F.B.C.); 2 Materiales Polifuncionales Basados en Carbono (UGR-Carbon), Departamento Química Inorgánica-Unidad de Excelencia Química Aplicada a Biomedicina y Medioambiente-Universidad de Granada (UEQ-UGR), ES18071 Granada, Spain; liliandr@correo.ugr.es (L.D.R.-V.); estherbg@ugr.es (E.B.-G.); fmarin@ugr.es (F.C.-M.); afperez@ugr.es (A.F.P.-C.)

**Keywords:** hydrochar, CQDs, agro-waste, TiO_2_, photocatalysis, visible light

## Abstract

This research aimed to co-produce CQDs and hydrochar from natural sources to improve the photocatalytic properties of TiO_2_. Juice extract from Citrus lemon fruits from south-eastern Spain was used as the carbon precursor. The synthesis strategy of the CQDs and hydrochar (Hc) was divided into different stages aimed at figuring out the role of the temperature (180, 220, 250 °C), the addition of TiO_2_ nanoparticles, and the presence of N-/P-donor compounds (ethylenediamine and orto-phosphoric acid) in the photocatalytic properties of final composites. The results revealed that at 250 °C, using agro-carbon materials as Hc, and the addition of N-donor compounds, improved the photocatalytic activity and photodegradation rate of TiO_2_ over methyl orange (MO) under blue light by 1000% and 2700%, respectively, with the parallel reduction of TiO_2_ bandgap from 3.5 eV (Uv light) to 3.00 eV (visible light). These results are related to the ability of the carbon materials (electronegative) to enhance the formation of a Ti^3+^-active state. This study provides a landscape for a one-step method for the production of agro-carbon/TiO_2_ photocatalysts with high activity under visible light as an efficient and sustainable strategy for applications such as energy generation and water purification under sunlight.

## 1. Introduction

Water contamination is one of the most hazardous environmental problems in the world and poses many risks to human life and the environment. Organic dyes are considered unsafe contaminants owing to their low degree of biodegradability and high toxicity. Therefore, they are considered to be among the most pernicious wastewaters [1]. Photocatalysis is widely used in decontamination processes because it uses ultraviolet (UV) or visible (VIS) light and a catalyst to accelerate chemical reactions that break down pollutants [2]. The mechanism is based on the ability of certain materials, called photocatalysts, to activate a chemical reaction. When the photocatalyst is exposed to light, photons of the light (usually ultraviolet) excite electrons in the material, raising them to a higher energy state. This leaves vacancies in the lower energy levels of the material, known as “holes”. The excited electrons and holes generated in the photocatalyst can interact with water or oxygen molecules present in the environment, resulting in the formation of reactive oxygen species (ROS) such as hydroxyl radicals (OH●) and hydrogen peroxide (H_2_O_2_) and free electrons that attack contaminants (organic or inorganic), breaking them down into simpler and less toxic compounds, such as carbon dioxide (CO_2_) and water (H_2_O) [3]. Titanium dioxide (TiO_2_) is one of the most studied photocatalysts because of its good photoelectronic properties, high stability, low cost, and non-toxicity. However, it has technical disadvantages such as limited efficiency in visible light, high rate of internal electron–hole recombination reduced ROS generation capacity, poor performance under real environmental conditions, problems with dispersion in liquid systems, photo-corrosion and deactivation over time, costs, and difficulties in the preparation of modified TiO_2_ materials [4]. Carbon quantum dots (CQDs), on the other hand, are a type of carbon nanomaterial with exceptional optoelectronic properties, due to the sp^2^ hybridization of their carbons, their amorphous or crystalline core, and their graphite lattice spacing [5]. CQDs have been reported as exceptional optoelectronic nanomaterials owing to their visible-light harvesting capabilities, tunable photoluminescence (PL), up-conversion photoluminescence and efficient transfer of photo-excited electrons [6]. They also have oxygenic functional groups (5–50% by weight) on their surface, which gives them water dispersibility and the possibility of functionalization. Depending on the synthesis methods used, the surface groups can be modified to further tune the PL by introducing an electron donor and/or acceptor. These conditions have allowed some researchers to focus on the photocatalytic capacity of CQDs, especially in the VIS and near-UV regions, overcoming the limitations of TiO_2_ [7]. Additionally, they can be produced from byproducts of the agroindustry, contributing to the concept of green chemistry [8]. Najjar et al. [9] synthesized CQDs from *Cordia Myxa* L. powder using a hydrothermal method at 180 °C for 4 h. The photocatalytic activity of the CQDs was evaluated during the degradation of Eriochrome Black T (EBT) dye under near-UV irradiation. The results revealed up to 100% EBT degradation in 40 min. A study conducted by Abd Rani et al. [10] employed biomass fruit bunches (FBs) and urea for the elaboration of nitrogen-doped carbon quantum dots (N-CQDs) by hydrothermal treatment at 180 °C for 8 h. Dye solutions in the presence of N-CQDs were exposed to UV near-irradiation at 302 nm, achieving 60–70% degradation between 10 and 30 min. Pemli et al. [11] synthesized CQDs by a hydrothermal method at a mild temperature (120 °C) using watermelon rinds. The authors evaluated the photocatalytic activity of the CQDs on the photodegradation of methyl orange (MO) dye using a reactor equipped with a halogen lamp (500 W) and VIS light (≅420 nm). The results revealed degradation of up to 65% after 120 min. Many researchers have demonstrated the photocatalytic benefits of the CQDs from agro-industrial waste [8,12,13], and this research will also demonstrate such benefits. However, to the best of our knowledge, though there is extensive literature on CQDs in photocatalysis, few studies use byproducts obtained during the synthesis of CQDs which, by their nature, can also present important physicochemical properties at a lower cost given their yield (>90%) [14]. During the hydrothermal synthesis of CQDs, the byproducts generated can vary depending on specific process conditions, such as temperature, reactant concentration, and reaction time [15]. Some authors have reported the generation of hydrochar, an insoluble phase consisting of amorphous carbon or graphite [16,17], and formic, acetic, lactic, and oxalic acids, dispersed in the water [18,19,20]. Owing to the carbonaceous nature of the hydrochar, it can exhibit photocatalytic properties that need to be evaluated [21]. This article explores the design of photocatalysts based on carbon structures obtained from the hydrothermal synthesis and both hydrochar and CQDs, which, in combination with TiO_2_, result in a viable strategy for photocatalysis under visible conditions. The combination of carbon-based optoelectronic structures can significantly improve the efficiency of TiO_2_ photocatalysis because they can alter the electron movement during catalysis by improving light absorption, reducing electron–hole recombination between Ti-atoms, and facilitating charge transfer, thus extending the photocatalytic activity to visible light.

## 2. Results and Discussion

**Figure 1:**
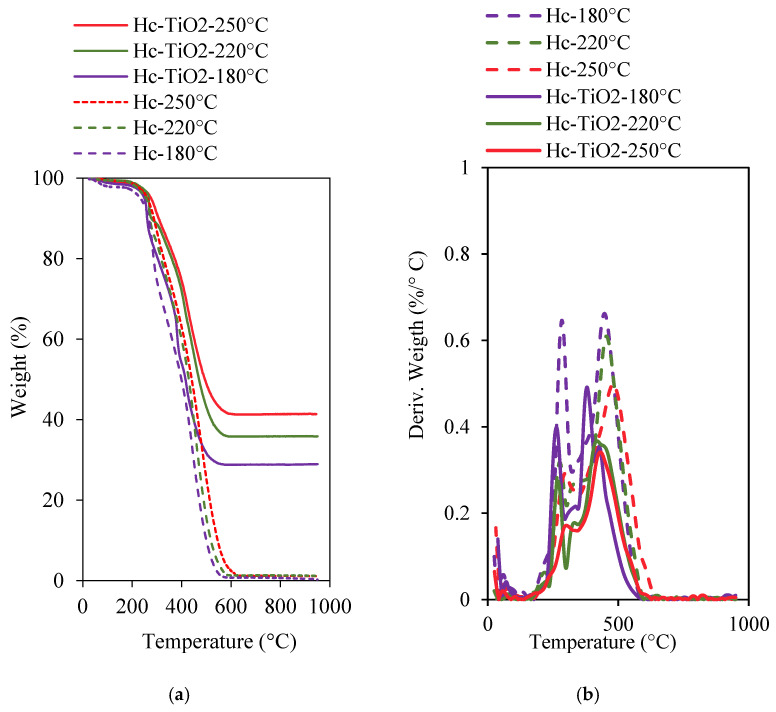
(**a**) Thermogravimetric analyses (TGA) and (**b**) derivative thermogravimetric curves (dTGA) for hydrochar and hydrochar–TiO_2_ composites synthetized at different temperatures. The curves were performed in triplicate (*n* = 3), and in no case did the statistical errors exceed 5%. The average measurement curve is reported.

To determine the amount of TiO_2_ chemisorbed on the hydrochar, thermogravimetric analyses were performed, as shown in Figure 1a. Gravimetric profiles allowed us to conclude that the TiO_2_ concentrations were 28.7%, 35.7%, and 41.2% for Hc-TiO_2_-180 °C, Hc-TiO_2_-220 °C and Hc-TiO_2_-250 °C, respectively. The correlation between the synthesis temperature and TiO_2_ content on Hc is explained by the greater reactivity of the functional groups of the carbon (Hc)–metal (Ti) pair and better diffusion of TiO_2_ throughout the hydrochar for the functionalization [22]. The results revealed an improvement in the thermal stability of the hydrochar in the presence of TiO_2_, which can be explained by the physical barrier properties offered by nanoparticles, as well as the ability to improve heat distribution in the hydrochar (thermal conductivity), and the capacity to prevent oxidation of organic structures at high temperatures [23,24]. Similarly, the dTGA curves (Figure 1b) of the Hc materials showed two main peaks associated with the decomposition of volatile matter at 200–400 °C and the oxidation of the carbonaceous structure at (400–550 °C) [23].

The results revealed that the increase in the hydrothermal synthesis temperature reduced the VM content in the Hc structure and increased the thermal stability of the carbonaceous material, as reported by other authors [24]. In the presence of TiO_2_ nanoparticles, the reduction in the VM content in Hc was more pronounced by the formation of the carbon (Hc)–metal (Ti) pair.

**Figure 2:**
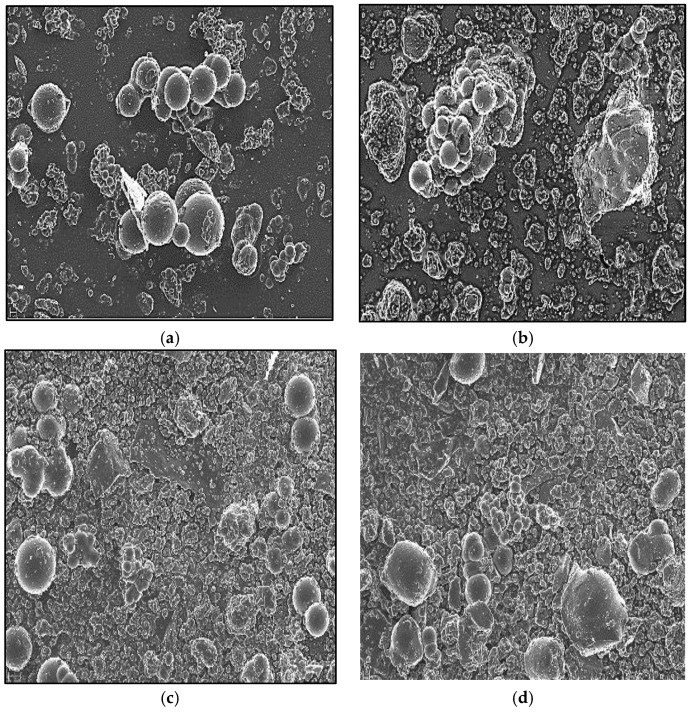
Field emission scanning electron microscopy (FE-SEM) images for (**a**) H_C_, (**b**) Hc-TiO_2_-180 °C, (**c**) Hc-TiO_2_-220 °C, and (**d**) Hc-TiO_2_-250 °C. Magnification: 5.00 K.

The Hc-TiO_2_ microstructure was evaluated using FE-SEM. Figure 2a shows spherical assemblies of micrometric order (>1 µm) corresponding to the primary particles of the hydrochar control (Hc). However, the Hc-TiO_2_ composites in Figure 2b–d show both primary particles of Hc, as well as agglomerates of smaller structures associated with TiO_2_. Other authors have reported this type of arrangement for carbon–TiO_2_ systems [25,26].

**Figure 3:**
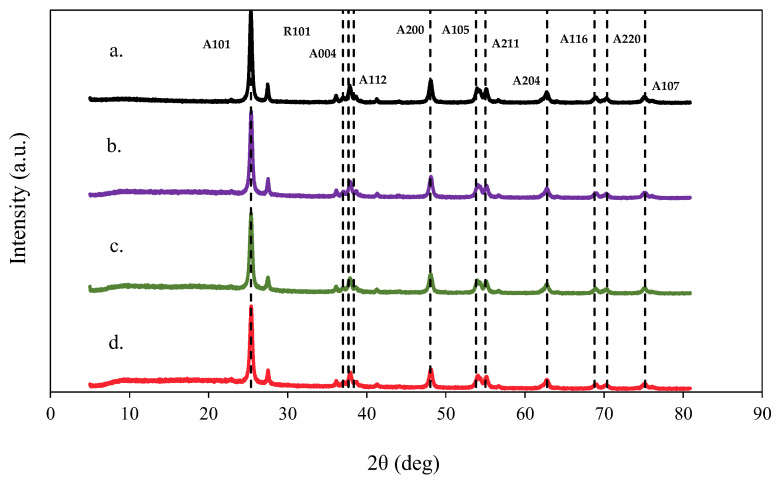
XRD pattern of (**a**) TiO_2_, (**b**) Hc-TiO_2_-180 °C, (**c**) Hc-TiO_2_-220 °C, and (**d**) Hc-TiO_2_-250 °C. The curves were calculated in triplicate (*n* = 3), and in no case did the statistical errors exceed 5%. The average measurement curve is reported.

Figure 3 shows the X-ray diffractograms of the TiO_2_ and Hc-TiO_2_ composites. The hc-based samples did not exhibit characteristic peaks related to the graphite phase content, supporting the idea of amorphous carbon in this type of waste. However, all Hc-TiO_2_ composites showed the characteristic peaks of the anatase; in particular, characteristic profiles of the rutile phase were observed, which suggests that the synthesis conditions caused a transformation towards this phase associated with the improvement of the catalytic performance of the materials [27].

The average crystal size (D) of the system was determined using the Debye–Scherrer equation [28], and the results are shown in Table 1. The D value decreased by up to 10% when TiO_2_ was chemisorbed onto the carbonaceous structures. The decrease in the size of TiO_2_ could be due to the surface reduction of titania during the hydrothermal treatment. The generation of oxygen vacancies as the temperature increases generates an amorphous surface that causes a reduction in the size of TiO_2_ crystals. Other authors have also reported this phenomenon [29].

**Figure 4:**
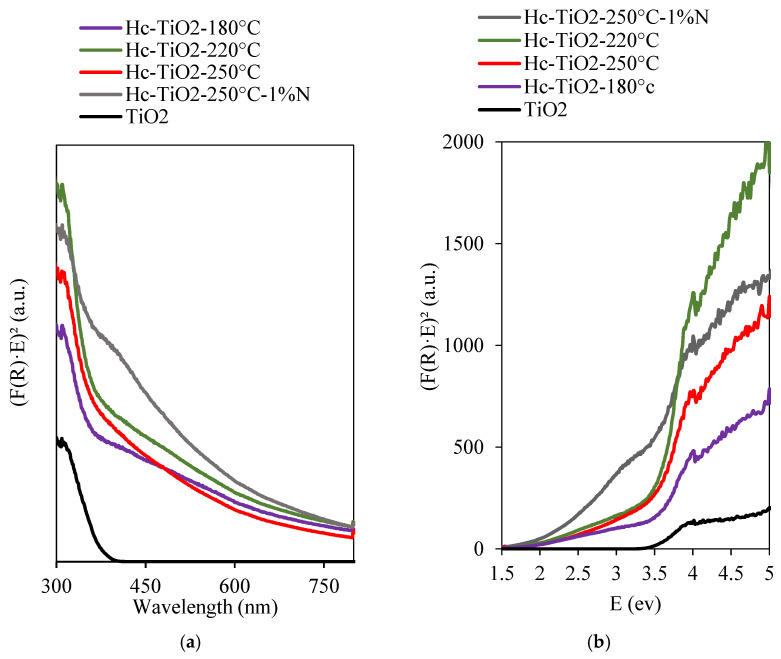
Diffuse reflectance spectra of TiO_2_, Hc-TiO_2_-180 °C, Hc-TiO_2_-220 °C, Hc-TiO_2_-250 °C and Hc-TiO_2_-250 °C-1%N based on (**a**) light absorption wavelengths and (**b**) energy expressed in electron volts. The curves were performed in triplicate (*n* = 3), and in no case did the statistical errors exceed 5%. The average measurement curve is reported.

The absorption spectra of the TiO_2_ and Hc-TiO_2_ samples are shown in Figure 4a. The Hc-TiO_2_ samples exhibited absorption edges around 550 nm rather than the TiO_2_ nanoparticles (<400 nm). Hence, the as-prepared composites were photoactive in the visible-light region, suggesting a clear improvement in the optical properties compared to the raw TiO2 nanoparticles. Figure 4b shows the (F(R)hυ)2 vs. hυ graph used to estimate the bandgap (BG) of the samples. The BG is the intersection of the extension of the linear portion of the curves with the *x*-axis. The results for TiO_2_, Hc-TiO_2_-180 °C, Hc-TiO_2_-220 °C, Hc-TiO_2_-250 °C, and Hc-TiO_2_-250 °C-1%N were 3.50, 3.43, 3.48, 3.35, and 3.00 eV, respectively. The Hc-TiO_2_ bandgap was lower than the of pure TiO_2_ owing to the interaction between the carbon and Ti-phases during carbonization. TiO_2_ is an intrinsic n-type semiconductor with a bandgap of approximately 3.2 eV. However, the higher density of the reduced Ti^3+^ state (more catalytic), as observed in the XPS analysis, may be responsible for the narrowing of the bandgap. This reduced form of Ti is associated with thermal synthesis in the presence of Hc as an electron acceptor [29].

Visible light on the solar spectrum has a wavelength between 400 nm and 700 nm; this corresponds to photon energies of approximately 1.77 eV to 3.26 eV, indicating that a system like Hc-TiO_2_-250 °C-1%N (3.00 eV) could usefully exploit visible light during photocatalytic processes. The results are optimistic, considering that a smaller bandgap makes it easier for electrons to be excited; that is, to move from the valence band to the conduction band with less energy, making TiO_2_ more efficient as a photocatalyst in the visible region [30,31].

**Figure 5:**
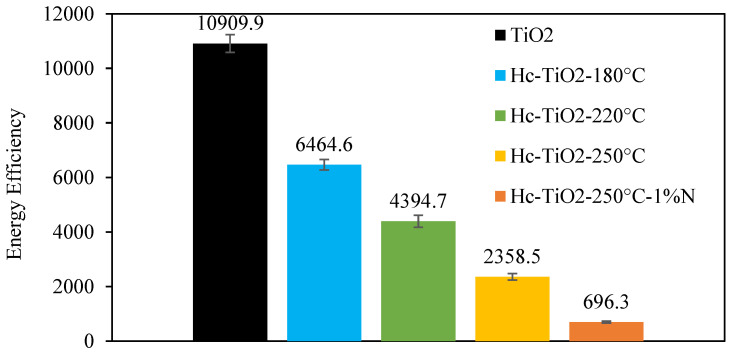
Energy efficiency (EEO) quantification for the photodegradation of MO under visible light using TiO_2_ and the as-prepared Hc-TiO_2_.

Figure 5 presents the energy efficiency (EEO) quantification for the photodegradation of MO under visible light using TiO2 and as-prepared Hc-TiO_2_.

The marginal photodegradation activity of TiO_2_ under visible light leads to high energy consumption (10,910 kWhm^−3^). However, the surface modification of TiO_2_ due to the formation of Hc on the nanoparticle surface reduces the energy consumption by more than 78% for the Hc-TiO_2_-250 °C composite (2358 kWhm^−3^). Furthermore, the incorporation of the N-donor during the CQD hydrothermal synthesis decreased energy consumption by 93% (696 kWhm^−3^). The main goal of this study was the co-production of Hc-TiO_2_ as a subproduct of CQD hydrothermal synthesis, making the use of TiO_2_ composites viable under visible light. In further studies, it is expected that researchers will include a comprehensive analysis to help estimate the quantum efficiency of MO photodegradation using the Hc-TiO_2_-250 °C-1%N sample.

### 2.1. Photodegradation of MO Using Hc-TiO_2_ Composites

#### 2.1.1. Effect of the Hydrochar and Temperature Synthesis

**Figure 6:**
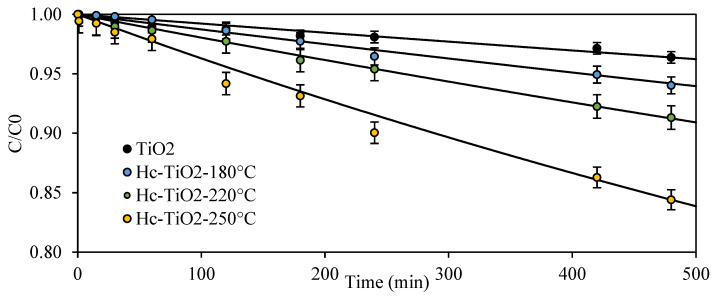
Photodegradation of MO using TiO_2_ and Hc-TiO_2_ composites synthetized at different temperatures. The second-order model (continuous lines) is shown to compare the theoretical and experimental trends. The curves were calculated in triplicate (*n* = 3), and in no case did the statistical errors exceed 5%. The average measurement curve is reported along with the error bars.

Figure 6 shows the photodegradation kinetics of MO at 465 nm in the presence of TiO_2_ and Hc-TiO_2_ composites synthesized at temperatures between 180 °C and 250 °C. TiO_2_ exhibited marginal photocatalytic activity with a value close to 3.6%. However, the photocatalytic activities of the Hc-TiO_2_ photocatalysts obtained at 180 °C, 220 °C, and 250 °C increased to 5.9, 8.7, and 15.6%, respectively.

Table 2 lists the parameters obtained by fitting the degradation kinetics of Figure 5 to the second-order models. According to the k parameter, the photodegradation of TiO_2_ improved with the presence of Hc and with an increase in the synthesis temperature, and the photodegradation rate improved by up to 400% when Hc-TiO_2_-250 °C was used instead of TiO_2_.

**Figure 7:**
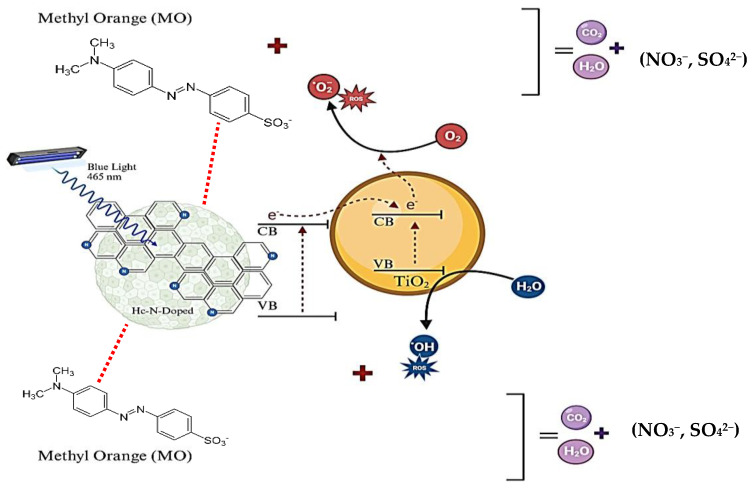
Proposed mechanism for MO photocatalysis using Hc-TiO_2_-based catalysts under visible light (465 nm).

In conclusion, synesthetic behavior between TiO_2_ and the carbon phase is inferred, especially at high synthesis temperatures where the amount of TiO_2_ is higher. The hydrochar influenced the bandgap of TiO_2_, which implies an improvement in photocatalytic efficiency, even at energy levels that are not characteristic of TiO_2_ (465 nm). Some authors have demonstrated that normally conjugated carbon structures (sp2–carbon) can significantly improve the efficiency of TiO_2_ photocatalysis because they can modify the electron movement (Ti^2+^ → Ti^3+^), reduce electron–hole recombination between Ti-atoms, and ease electron transfer, extending the photocatalytic activity of TiO_2_ to visible light [7,32,33], as evidenced in the present investigation. Some authors have also reported that carbon-based materials can provide a surface for the degradation of organic contaminants, favoring TiO_2_-molecule contact during photocatalysis [34]. A possible mechanism for this phenomenon is shown in Figure 7.

#### 2.1.2. Effect of the Heteroatom Donor During Hydrochar Synthesis

**Figure 8:**
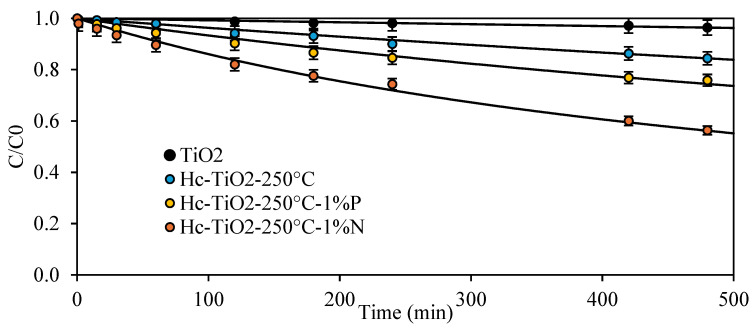
Photodegradation of MO using TiO_2_ and Hc-TiO_2_-250 °C synthetized in the presence of N/P donors. The second-order model (continuous lines) is shown to compare the theoretical and experimental trends. The curves were calculated in triplicate (*n* = 3), and in no case did the statistical errors exceed 5%. The average measurement curve is reported along with the error bars.

Figure 8 shows the photodegradation kinetics of MO for Hc-TiO_2_-250 °C, the best photocatalyst, synthesized in presence of N/P donors. The photocatalytic activity of the Hc-TiO_2_-250 °C increased from 15.6% to 43.7% and from 15.6% to 24% using N donors and P donors, respectively.

Table 3 lists the parameters obtained by fitting the degradation kinetics from Figure 7 to the second-order models. Based on the k parameter, the photodegradation capacity of HC-TiO_2_-250 °C in the presence of N-donor was significantly improved, achieving photodegradation rate increases of up to 2700% when Hc-TiO_2_-250 °C-1%N was used instead of TiO_2_. That is, the use of N-doped composites (Hc-TiO_2_-250 °C-1%N) improved the photocatalytic capacity of TiO_2_ seven times more than its un-doped analogues (Hc-TiO_2_-250 °C).

The improvement in the photocatalytic performance of the Hc-TiO_2_-250 °C with the addition of nitrogenous groups is related to the reduction in the photocatalyst bandgap and better use of the light source, as shown in Figure 4. Some authors have shown that this reduction in the energy gap allows nitrogen-doped carbonaceous materials to absorb and emit light over a wider wavelength range, particularly in the visible-light region [35,36,37]. The reduction in the bandgap is associated with the generation of active states of Ti (Ti^3+^) in the presence of non-metallic heteroatoms [29]. Other authors have shown that the functional groups introduced by nitrogen improve the dispersion of carbonaceous materials in a solution, refining the stability and distribution of the structures during their action [38]. Finally, it has been reported that nitrogen doping reduces the non-radiative recombination of electrons and holes generated during optical excitation [39]. Nitrogen acts as a trap center for electrons, which can improve the efficiency of photoluminescence by reducing the energy loss during the emission process [40].

### 2.2. Photodegradation of MO Using Co-Produced CQDs

**Figure 9:**
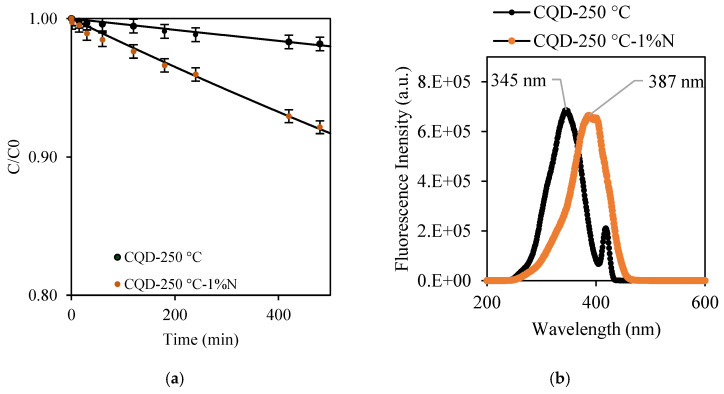
Photodegradation of MO using (**a**) CQD-250 °C and CQD-250 °C-1%N. The second-order model (continuous lines) is shown to compare the theoretical and experimental trends. (**b**) Excitation spectrum for CQD-250 °C and CQD-250 °C-1%N. The curves were performed in triplicate (*n* = 3), and in no case did the statistical errors exceed 5%. The average measurement curve is reported along with the error bars.

The CQDs co-produced during the synthesis of Hc-TiO_2_-250 °C and Hc-TiO_2_-250 °C-1%N were isolated and their photodegradation capacity at 465 nm was evaluated. The maximum photodegradation in the presence of CQD-250 °C and CQD-250 °C-1%N at 400 mg/L was 1.8 and 7.9%, respectively (Figure 9a). The incorporation of nitrogen into the CQD structure enhanced its photocatalytic activity. This improvement can be attributed to the greater excitation of CQD-250 °C-1%N compared to CQD-250 °C in the visible region, as evidenced by the fluorescence spectra (Figure 9b). The maximum excitation wavelength for CQD-250 °C was observed at 345 nm, while for CQD-250 °C-1%N, it shifted to 387 nm. This redshift allowed for more effective utilization of blue light during photocatalysis with CQD-250 °C-1%N, and resulted in an approximately 0.06 EJ reduction in excitation energy—an 11% decrease—based on Planck’s energy equation.

**Figure 10:**
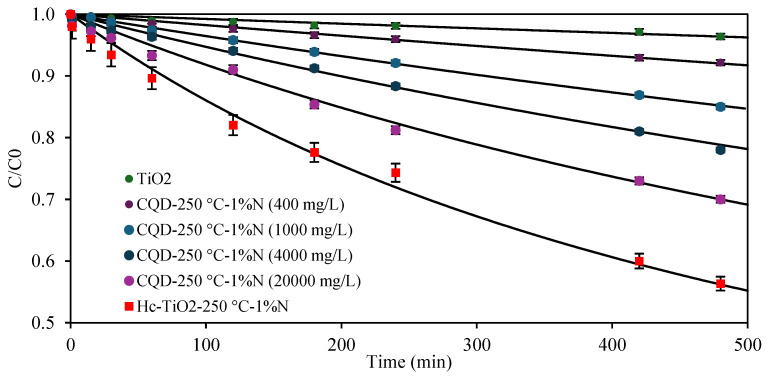
Photodegradation of MO using CQD-250 °C-1%N at concentrations between 400 mg/L and 20,000 mg/L. The second-order model (continuous lines) is shown to compare the theoretical and experimental trends. The curves were calculated in triplicate (*n* = 3), and in no case did the statistical errors exceed 5%. The average measurement curve is reported along with the error bars.

However, the photocatalytic performance of CQD-250 °C-1%N was lower than those obtained for the co-product Hc-TiO_2_-250 °C-1%N, which may be due to the concentration of the photocatalysts used, 400 mg/L vs. 2427 mg/L, and the carbon–Ti synergy of the Hc-TiO_2_ composite. To review the first hypothesis, photocatalytic tests were carried out at higher concentrations of CQD-250 °C-1%N, between 400 and 20,000 mg/L, and the results are shown in Figure 10.

The results showed a positive correlation between the concentration and the photocatalytic capacity of CQD-250 °C-1%N, but the systems based on Hc-TiO_2_ continued to show better performances. These differences can be attributed to the abundance of carbon arrangements of different sizes, structures, and compositions in Hc [41,42], which can enhance the optoelectronic performance of the Hc-TiO_2_, as well as the presence of an intrinsic catalyst such as TiO_2_. Despite these differences, both CQD-250 °C-1%N and Hc-TiO_2_-250 °C-1%N achieved better photocatalytic performances than those revealed by TiO_2_ at the same concentration. Table 4 shows the parameters obtained by fitting the degradation kinetics of final systems, such as TiO_2_, CQD-250 °C-1%N, and Hc-TiO_2_-250 °C-1%N, to second-order models. Based on the k parameter, the capacity of TiO_2_ is marginal; however, photocatalysts obtained during a single synthesis can improve the TiO_2_ photocatalytic rate from 476% to 2700% using CQDs and Hc, which translates into better utilization of the visible radiation used.

**Figure 11:**
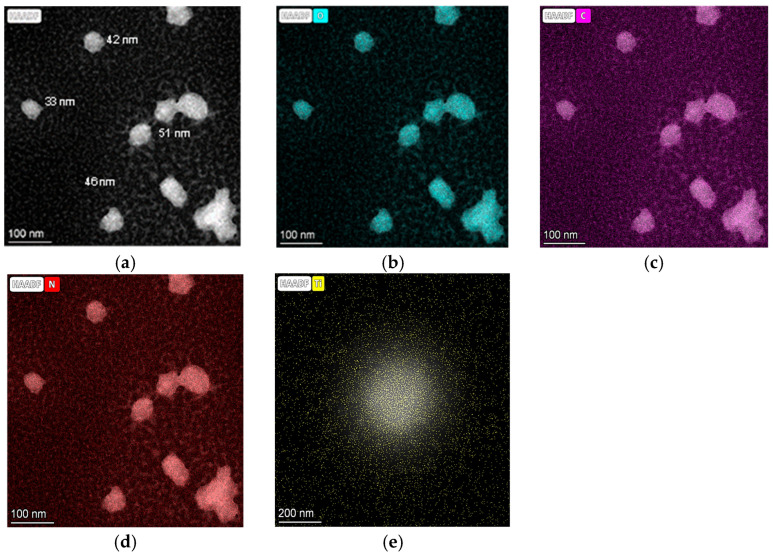
HR-TEM elemental mapping images for CQD-250 °C-1%N synthetized in the presence of TiO_2_ for visualization of atoms of (**a**) hydrogen (white), (**b**) oxygen (cyan), (**c**) carbon (violet), (**d**) N (red), and (**e**) Ti (yellow) atoms.

The proposed hydrothermal synthesis protocol allowed the chemisorption of TiO_2_ on the hydrochar structure but not on the CQDs. Figure 11 shows the HR-TEM elemental mapping images for CQD-250 °C-1%N. The results revealed the occurrence of various atoms, including carbon (violet), oxygen (cyan), and N (red) on the CQDs nanoparticles; however, the TiO_2_ mapping image did not reveal the presence of Ti (yellow) chemisorbed onto the CQD nanostructures, as was observed for the other atoms; instead, the atoms were dispersed throughout the field of view.

The absence of chemisorbed TiO_2_ could explain the weaker photocatalytic performance of CQDs compared to Hc-TiO_2_ composites rich in Ti-derived species, as verified by TGA and bandgap analysis. The combination of optoelectronics structures such as N-modified Hc and TiO_2_ can significantly improve efficiency and expand the ability of photocatalytic systems to operate under visible light, especially because carbonaceous structures with sp^2^-hybridization have the capacity to enhance electronic movements and generate more active forms of Ti and oxygen [7,43,44].

**Figure 12:**
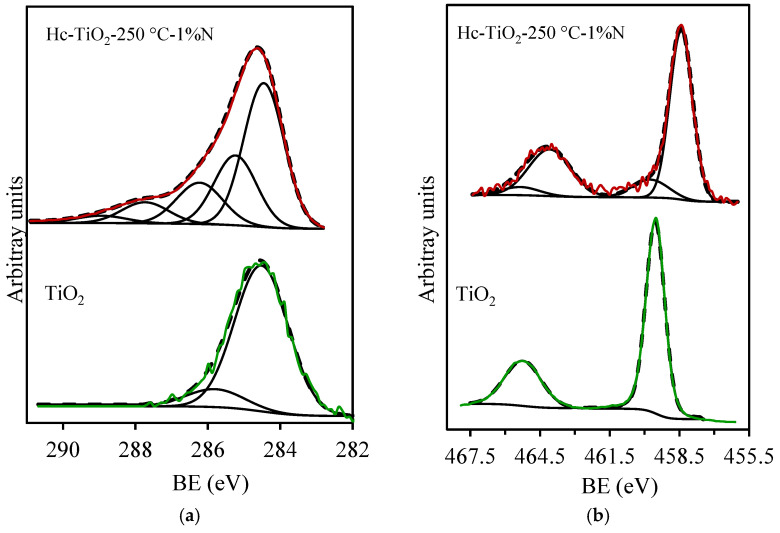

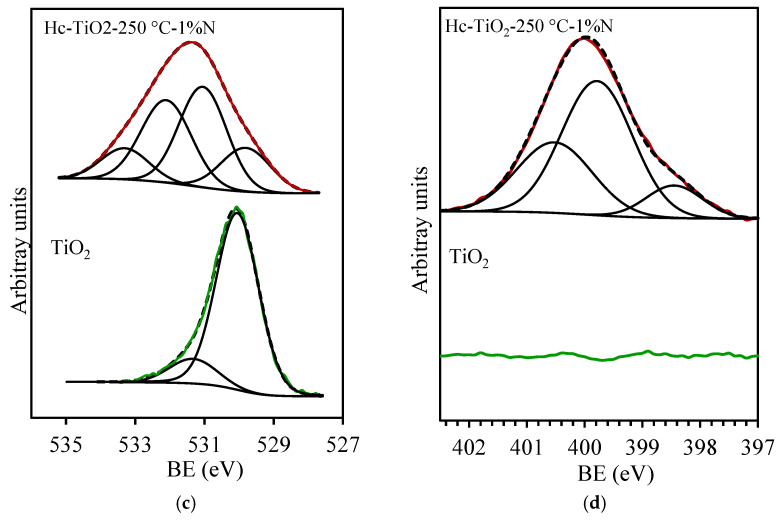
XPS spectra of TiO_2_ and Hc-TiO_2_-250 °C-1%N. (**a**) C_1s_, (**b**) Ti_2p_, (**c**) O_1s_, and (**d**) N_1s_ regions.

Finally, the superiority of Hc-TiO_2_-250 °C-1%N during photocatalysis can also be explained. Figure 12 shows the surface composition of this photocatalyst obtained by XPS. The results display the C_1s_ region for TiO_2_ and Hc-TiO_2_-250 °C-N samples. The peak at 284.6 eV was assigned to the C-C bond and used as an internal reference to correct the binding energy shift. The peaks at higher binding energies were attributed to the presence of oxygen-containing surface groups. In the TiO_2_ sample, the detected C peak in the spectrum was attributed to the adventitious carbon atoms adsorbed on the TiO_2_ surface. However, a better-defined region was observed in the Hc-TiO_2_-250 °C-1%N sample due to the presence of the hydrochar. The Ti_2p_ spectral region (Figure 12b) of the TiO_2_ sample presents only one component at BE = 459.6 eV, corresponding to Ti^4+^, in agreement with previously published BE values [34]. However, in the case of the Hc-TiO_2_-250 °C-N sample, two peaks are observed: the first one, at 459.6 eV, corresponds to Ti^4+^, and the peak at 458.4 eV is assigned to Ti^3+^. Note that 85.7% of surface titania is in a 3+ oxidation state, indicating the highly defective nature of the titania surface in the Hc-TiO_2_-250 °C-1%N sample, as has been proposed. The high content of Ti^3+^ or oxygen vacancies on the titania surface could explain the significant decrease in the bandgap in this sample and, consequently, the better photoactivity.

Two components, at 530.1 and 531.3 eV, were used to fit the O_1s_ spectral region of TiO_2_ samples (Figure 12c). The first component, which is the major component of the O_1s_ spectral region, corresponds to lattice oxygen (OL), whereas the high BE component corresponds to oxygen-containing species adsorbed in oxygen vacancies (OVs) [45]. However, four components were used to fit the O_1s_ spectral region of Hc-TiO_2_-250 °C-1%N sample because of the formation of different Ti-species and the oxygen linked to the carbon phase. Thus, as previously mentioned, the component at 530.1 eV corresponds to oxygen bonded to Ti^4+^, whereas oxygen bonded to Ti^3+^ appears at 531.3 eV. In this case, the OV peak was the major component of the TiO_2_ sample, confirming the highly defective nature of the titania surface observed in the Ti_2p_ region. It is important to note that the OVs in metal oxides play a crucial role in the generation of reactive oxygen species (ROS) because of their ability to store and release electrons. When these materials encounter molecular oxygen (O_2_), the electrons located in the vacancies can be transferred to oxygen, initiating a sequence of redox reactions that give rise to various ROS. In the first step, molecular oxygen accepts an electron to form a superoxide ion (O_2_⁻), which can then be protonated to generate the hydroperoxide radical (HO_2_•). As electron and proton transfer continue, species such as hydrogen peroxide (H_2_O_2_) and, eventually, the hydroxyl radical (•OH), one of the most reactive ROS, are formed. This mechanism underscores how structural defects in materials with oxygen vacancies not only alter their electronic properties but also transform them into efficient platforms for oxidative processes in applications such as environmental photocatalysis [45].

On the other hand, the peaks at 532.4 and 533.6 eV correspond to the oxygenated surface groups of the carbon phase, which are assigned, respectively, to C=O and C-O bonds. Finally, the N_1s_ region is depicted in Figure 11d. Note that N was not detected in the TiO_2_ sample, whereas up to 4 wt.% was detected in the Hc-TiO_2_-250 °C-1%N sample, denoting its high surface N-doping content. Three components, at 598.4, 399.8 and 400.5 eV, were used to fit the N_1s_ spectral region attributed to Pyridinic, Pyrrolic and graphitic N-groups anchored on the carbon surface. It has also been also reported that the XPS peaks at 396–397 eV are due to substitutional N-atoms, and those that appear at approximately 400 eV are generally ascribed to interstitial nitrogen in TiO_2_ [45,46]. In our work, peaks detected at 398.4 and 399.8 eV can also be attributed to the substitutional and interstitial states of nitrogen. Therefore, N-doping of TiO_2_ could occur in the hydrochar-based materials, which could explain the highly defective surface and the performance of Hc-TiO_2_-250 °C-1%N.

Finally, others’ research has shown that by using a sensitized photocatalyst, such as carbon- or nitrogen-doped TiO_2_, the photochemical properties can be further improved due the generation of electronegative sites that can extend the material’s ability to generate ROS under visible-light conditions for the decomposition of polluting organic compounds and other oxidation reactions [47,48].

## 3. Materials and Methods

### 3.1. Chemicals

*Citrus lemon* fruits were collected in south-eastern Spain. Titania, TiO_2_ anatase—hereinafter TiO_2_ (Sigma-Aldrich, St. Louis, MO, USA)—was used as the reference photocatalyst. Ethylenediamine (C_2_H_8_N_2_) (99%) (PanReac, Barcelona, Spain) and 85% Orthophosphoric acid (H_3_PO_4_) (Sigma-Aldrich, St. Louis, MO, USA) were used as nitrogen donors (N-donors) and phosphorous donors (P-donors), respectively. Methyl Orange (MO, C_14_H_14_N_3_NaO_3_S) was procured from Acros Organics (Brussels, Belgium) and used as a pollutant during the photocatalysis assays. The water used was purified using a Millipore instrument.

### 3.2. Co-Production of Hydrochar and CQDs Nanoparticles

The synthesis strategy was divided into different steps to determine the role of the temperature, the addition of TiO_2_, and N-/P-donor inclusion on the photocatalytic properties of the hydrochar (Hc) and CQDs obtained by the hydrothermal process. A one-step hydrothermal carbonization method was used to produce carbon structures from natural sources [49,50]. *Citrus lemon* fruits produced in south-eastern Spain were washed, cut, and then squeezed to obtain the juice extract (LJ) used as the carbon precursor owing to its high organic acid content [51]. Lemon juice was prepared in the absence or presence of 0.5 g of TiO_2_, then magnetically stirred, and finally transferred into a 200 mL Teflon-lined stainless-steel autoclave. The hydrothermal treatment was conducted by varying the temperature synthesis from 180 to 250 °C for 6 h, and the autoclave was cooled down to room temperature. The obtained solution was filtered through a 0.22 µm filter membrane to remove the solid phase or hydrochar from the aqueous dispersion of the CQDs. The collected hydrochar was washed several times using deionized water at 70 °C to promote the desorption of residual CQD structures on the surface. The aqueous dispersions of CQDs were concentrated to 50 mL in a drying oven at 70 °C. For heteroatoms-doped CQDs, ethylenediamine (C_2_H_8_N_2_) and Orthophosphoric acid (H_3_PO_4_) were used as nitrogen donors (N-donors) and phosphorous donors (P-donors), respectively [52,53]. To this end, the N-/P-donor compound was added to the lemon juice at a mass concentration of 1%wt before the inclusion of TiO_2_ and afterwards, the described procedure was conducted. The convention used for the composites obtained using LJ, TiO_2_, N/P donors at different temperatures was CS-TiO_2_-T°C-1%HETAM, where CS = Hc or CDQ, T = 180 °C, 220 °C or 250 °C, and HETAM = N or P. For example, Hc-TiO_2_-250 °C-1%N indicates that Hc/TiO_2_ composites were obtained at 250 °C using 1% of N-donor.

### 3.3. Characterization of Carbon Structures

The thermal stability and the TiO_2_ amount on hydrochar were studied under a specific temperature range (34–950 °C) using thermogravimetric analysis (Mettler-Toledo International Inc., Greifensee, Switzerland). The temperature was increased by passing nitrogen at ramping rate of 5 °C/min and a flow rate of 40 mL/min. Field emission scanning electron microscopy (FE-SEM Carl Zeiss, Jena, Germany) was employed to examine the morphology of the hydrochar-based composites. An X-ray diffractometer (Bruker D8) with Cu Kα radiation and a wavelength (λ) of 1.541 Å was used to determinate the crystallographic phases of the samples. The bandgap of the samples was estimated using UV-Vis diffuse reflectance spectroscopy (CARY 5E from VARIAN) [54]. Finally, High-Resolution Transmission Electron Microscopy (HR-TEM) was performed using a Carl Zeiss SMT LIBRA 120 Plus microscope (Carl Zeiss, Jena, Germany) to evaluate the morphology of the CQDs. Finally, X-ray photoelectron spectroscopy (XPS) was performed using a Kratos Axis Ultra-DLD spectrometer (Dallastown, PA, USA) equipped with a hemispherical electron analyzer connected to a delay-line detector (DLD), a dual-anode X-ray source (Mg/Al) with a power output of 450 W, and an Al Kα monochromator with a nominal power of 600 W, adjusting the spectra to Lorentzian and Gaussian curves.

### 3.4. Photocatalytic Trials

**Figure 13:**
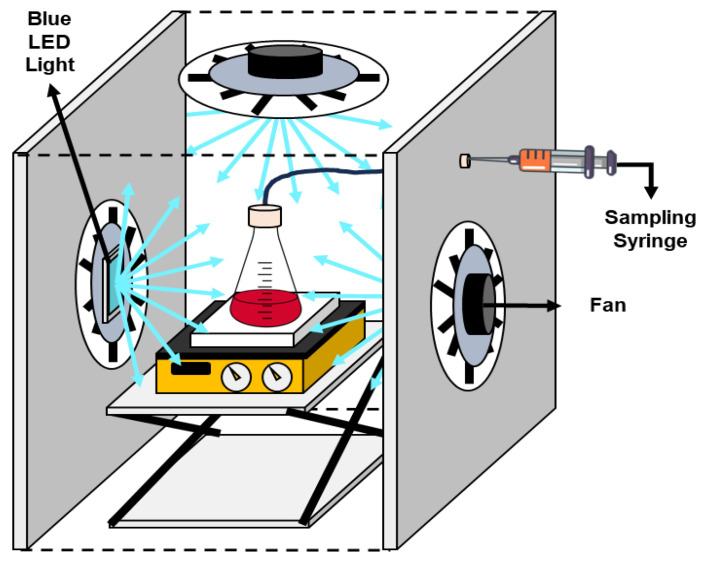
Schematic representation of the experimental setup for the photocatalytic test.

The performance during the degradation of Methyl Orange (MO) under visible-light irradiation at 30 °C was evaluated in a reaction system composed of a borosilicate glass reactor, a sampling syringe, and two power LEDs (Blue LED light at 465 nm, electric power of 50 W and 4080 lumen/W) coupled with fan devices located above and on the sides of the glass reactor [55] as shown in Figure 13. The pH of the MO solutions was adjusted to 7 using NaOH (0.01 N) and HCl (0.01 N) solutions before the assay.

The photodegradation of MO was performed in the reactor described using 100 mL of a solution containing 1 mg of MO, 100 mg of TiO_2_ and adjusted Hc-TiO_2_ concentrations that could guarantee an equal presence of TiO_2_ in Hc-TiO_2_ systems [29]. The suspensions were stirred at 500 rpm in the dark for 12 h until equilibrium was reached. The reactor was illuminated using Blue LED Light at 465 nm. Every 30–50 min for 500 min, samples were withdrawn and centrifuged to separate the nanoparticles and residual MO was measured by spectrophotometry at 464 nm (6505 JENWAY, London, UK) and a previously calibrated curve. The % of MO photodegradation (% MO) was calculated using Equation (1) [54].

(1) $$\%\;of\;MO=\left[\frac{C_{0}-C_{t}}{C_{0}}\right]\times 100$$

where $C_{0}$ is the initial concentration of MO and $C_{t}$ is the concentration after irradiation at a specific time. The MO photodegradation kinetics were adjusted using a second-order kinetic model according to Equation (2).

(2) $$\frac{1}{C_{t}}=k\times t+\frac{1}{C_{0}}$$

where $C_{0}$ is the initial MO concentration, $C_{t}$ is the concentration after irradiation at a specific time (t), and k is the photocatalysis rate.

### 3.5. Electrical Energy per Order (EEO)

The Electrical Energy per Order (EEO) concept was used to quantify the energy efficiency of the photodegradation of MO under visible light using TiO_2_ and Hc-TiO_2_ composites using Equation (3) [54]:

(3) EEO=P×t×1000V×60×ln⁡C0Cf

where P is the power rate (kW) of the photoreactor system, V is the volume (L), C_0_ is the initial MO concentration, C_f_ is the final Mo concentration, and t is the irradiation time (min).

## 4. Conclusions

This study explored the effects of carbon materials, CQDs, hydrochar, and synthesis variables on the photocatalytic properties of TiO_2_. After the experiments, it is possible to conclude that a temperature of 250 °C, the presence of agro-carbon materials as hydrochar (52% wt), and the addition of N-donor compounds (1% wt) can improve the photodegradation rate of TiO_2_ over Methyl Orange (MO) by up to 2700%, with a parallel reduction in the TiO_2_ bandgap from 3.50 eV (Uv light) to 3.00 eV (visible light) associated with the development of abundant Ti^3+^ forms and oxygen vacancies. The development of photocatalysts based on agro-carbon–nitrogen-doped TiO_2_ is an efficient and sustainable strategy for applications such as power generation, water purification, and pollutant decomposition under sunlight. While this study focused on the systematic screening of modified TiO_2_ materials to identify promising photocatalysts, future work will include detailed evaluations of their stability and reusability over multiple cycles to assess their potential for practical applications.

## Figures and Tables

**Table 1:** Average crystal size (from XRD) of TiO_2_, Hc-TiO_2_-180 °C, Hc-TiO_2_-220 °C, and Hc-TiO_2_-250 °C photocatalysts.

Photocatalysts	D (nm)
TiO_2_	20.7
Hc-TiO_2_-180 °C	20.1
Hc-TiO_2_-220 °C	18.7
Hc-TiO_2_-250 °C	18.7

**Table 2:** Second-order kinetic parameters for the photodegradation of MO using Hc-TiO_2_ composites synthetized at different temperatures.

Photocatalysts	k × 10^5^ (L·mg^−1^·min^−1^)	%D	R^2^
TiO_2_	0.54	0.24	0.99
Hc-TiO_2_-180 °C	0.56	0.13	0.99
Hc-TiO_2_-220 °C	1.29	1.20	0.99
Hc-TiO_2_-250 °C	2.74	0.51	0.98

**Table 3:** Second-order kinetic parameters for the photodegradation of MO using Hc-TiO2-250 °C composites synthetized in the presence of N/P-donors.

Photocatalysts	k × 10^5^ (L·mg^−1^·min^−1^)	%D	R^2^
TiO_2_	0.54	0.24	0.99
Hc-TiO_2_-250 °C	2.74	0.51	0.99
Hc-TiO_2_-250 °C-1%P	3.21	0.84	0.99
Hc-TiO_2_-250 °C-1%N	15.57	1.40	0.98

**Table 4:** Second-order kinetic parameters for the photodegradation of MO using CQD-250 °C-1%N and Hc-TiO_2_-250 °C-1%N, obtained during a unique process of synthesis.

Photocatalysts	k × 10^5^ (L·mg^−1^·min^−1^)	%D
TiO2	0.54	0.24
CQD-250 °C-1%N	3.53	0.17
Hc-TiO2-250 °C-1%N	15.57	1.40

## Data Availability

The data are contained in the article.

## Abbreviations

The following abbreviations are used in this manuscript:

Hc	Hydrochar
MO	Methyl Orange
UV	Ultraviolet
VIS	Visible
ROS	Reactive oxygen species
TiO_2_	Titanium dioxide
OH●	Hydroxyl radicals
H_2_O_2_	Hydrogen peroxide
CQDs	Carbon quantum dots
PL	Photoluminescence
EBT	Eriochrome black t
N-CQDs	Nitrogen-doped carbon quantum dots
C_2_H_8_N_2_	Ethylenediamine
H_3_PO_4_	Orthophosphoric acid
N-donor	Nitrogen donor
P-donor	Phosphorous donor
HETAM	Heteroatom
FE-SEM	Field emission scanning electron microscopy
HR-TEM	High-resolution transmission electron microscopy
XPS	X-ray photoelectron spectroscopy
LJ	Juice extract
TGA	Thermogravimetric analysis
%MO	% of MO photodegradation
D	Average crystal size
nm	Nanometer
